# Comment: *Head and Neck Oncology*

**DOI:** 10.1186/1741-7015-12-24

**Published:** 2014-02-05

**Authors:** BioMed Central

**Affiliations:** 1 Floor 6, 236 Gray’s Inn Road, London WC1X 8HB, UK

## Abstract

This comment relates to articles published in archived content of the journal *Head and Neck Oncology* and is not related to the content of *BMC Medicine* in any way.

## Comment

This comment relates to articles published in archived content of the journal *Head and Neck Oncology* and is not related to the content of *BMC Medicine* in any way.

While conducting an internal audit of publications between January and June 2012, BioMed Central discovered a number of apparent major irregularities in the content and editorial handling of the journal *Head & Neck Oncology*. In order to maintain the integrity of the BioMed Central portfolio of journals, we decided to cease publication of the journal with effect from 9th August 2012.

University College London (UCL) and University College London Hospital (UCLH) subsequently conducted a joint investigation of our concerns. This focused primarily on the actions of Editor-in-Chief Mr Colin Hopper, because neither of the other active Editors-in-Chief, Waseem Jerjes and Tahwinder Upile, were employees of UCL during the time covered by our audit, so an investigation of their actions would have been beyond the scope of UCL’s investigation.

Following their investigation UCL were satisfied that there was no evidence of research misconduct arising from any employee, honorary researcher or student in relation to the articles they were asked to investigate. They were also satisfied that there was no evidence of editorial or author misconduct on the part of Colin Hopper.

In the absence of definitive conclusions about all of the concerns raised by its audit, BioMed Central has provided details of its findings in the form of a Publishers Note on relevant articles which will be updated if further information becomes available. If you are an author of a published article in this journal and have further questions, please contact info@biomedcentral.com.

The affected articles are listed below:

A review of the epidemiology of oral and pharyngeal carcinoma: update

Daniel M Saman

*Head & Neck Oncology* 2012, **4**:1

http://www.headandneckoncology.org/content/4/1/1

Randomized clinical trial of LigaSure versus conventional suture ligation in thyroid surgery

Anandi H.W. Schiphorst, Bas A. Twigt, Sjoerd G. Elias and Thijs van Dalen

Head & Neck Oncology 2012, 4:2

http://www.headandneckoncology.org/content/4/1/2

A decision support system for quality of life in head and neck oncology patients

Joaquim J Goncalves and Alvaro Rocha

Head & Neck Oncology 2012, 4:3

http://www.headandneckoncology.org/content/4/1/3

Etiology analysis and computed tomography imaging of a tonsillar inflammatory myofibroblastic tumor: report of an immunocompetent patient and brief review

Yun-Zhen Luo, Li-Bo Dai, Shui-Hong Zhou, Xing-Mei Luo, Jun Fan and Ling-Xiang Ruan

Head & Neck Oncology 2012, 4:4

http://www.headandneckoncology.org/content/4/1/4

cTNM vs. pTNM: the effect of not applying ultrasonography in the identification of cervical nodal disease

Waseem Jerjes, Tahwinder Upile, Hani Radhi, Aviva Petrie, Jesuloba Abiola, Aidan Adams, Jacqueline Callear, Panagiotis Kafas, Syedda Abbas, Kartic Rajaram, Colin Hopper

Head & Neck Oncology 2012, 4:5

http://www.headandneckoncology.org/content/4/1/5

The effect of tobacco and alcohol and their reduction/cessation on mortality in oral cancer patients: short communication

Waseem Jerjes, Tahwinder Upile, Hani Radhi, Aviva Petrie, Jesuloba Abiola, Aidan Adams, Panagiotis Kafas, Jacqueline Callear, Ramin Carbiner, Kartic Rajaram, Colin Hopper

Head & Neck Oncology 2012, 4:6

http://www.headandneckoncology.org/content/4/1/6

Nimesulide inhibited the growth of hypopharyngeal carcinoma cells via suppressing Survivin expression

Jia-Jun Tian, Su-Mei Lu, Liang Yu, Ju-Ke Ma, Ya-Kui Mu, Hai-Bo Wang and Wei Xu

Head & Neck Oncology 2012, 4:7

http://www.headandneckoncology.org/content/4/1/7

Primary squamous cell carcinoma of thyroid: a case report and review of literature

Mutahar A Tunio, Mushabbab Al Asiri, Mosa Fagih and Rashad Akasha

Head & Neck Oncology 2012, 4:8

http://www.headandneckoncology.org/content/4/1/8

The prognostic significance of p63 and Ki-67 expression in myoepithelial carcinoma

You-Hu Jiang, Bo Cheng, Ming-Hua Ge and Gu Zhang

Head & Neck Oncology 2012, 4:9

http://www.headandneckoncology.org/content/4/1/9

Parapharyngeal space hemangiopericytoma treated with surgery and postoperative radiation- a case report

Muhammad Mohsin Fareed, Abdullah Suleiman Mazaed Al Amro, Rashad Akasha, Mansour A Assiry, Mushabbab Al Asiri, Mutahar Tonio and Yasir Bayoumi

Head & Neck Oncology 2012, 4:10

http://www.headandneckoncology.org/content/4/1/10

Knockdown of aberrantly expressed nuclear localized decorin attenuates tumour angiogenesis related mediators in oral cancer progression model in vitro

Nyla Dil and Abhijit G Banerjee

Head & Neck Oncology 2012, 4:11

http://www.headandneckoncology.org/content/4/1/11

A retrospective, deformable registration analysis of the impact of PET-CT planning on patterns of failure in stereotactic body radiation therapy for recurrent head and neck cancer

Kyle Wang, Dwight E Heron, John C Flickinger, Jean-Claude M Rwigema, Robert L Ferris, Gregory J Kubicek, James P Ohr, Annette E Quinn, Cihat Ozhasoglu and Barton F Branstetter

Head & Neck Oncology 2012, 4:12

http://www.headandneckoncology.org/content/4/1/12

Clear cell chondrosarcoma of the head and neck

Sepideh Mokhtari and Abbas Mirafsharieh

Head & Neck Oncology 2012, 4:13

http://www.headandneckoncology.org/content/4/1/13

Delay in pathological tissue processing time vs. mortality in oral cancer: Short communication

Waseem Jerjes, Tahwinder Upile, Hani Radhi, Aviva Petrie, Aidan Adams, Jacqueline Callear, Panagiotis Kafas, Colin Hopper

Head & Neck Oncology 4:14

http://www.headandneckoncology.org/content/4/1/14

The cost burden of oral, oral pharyngeal, and salivary gland cancers in three groups: commercial insurance, medicare, and medicaid

Jed J Jacobson, Joel B Epstein, Frederick C Eichmiller, Teresa B Gibson, Ginger S Carls, Emily Vogtmann, Shaohung Wang and Barbara Murphy

Head & Neck Oncology 2012, 4:15

http://www.headandneckoncology.org/content/4/1/15

Photodynamic therapy in the management of potentially malignant and malignant oral disorders

Waseem Jerjes, Zaid Hamdoon, Colin Hopper

Head & Neck Oncology 4:16

http://www.headandneckoncology.org/content/4/1/16

CO2 lasers in the management of potentially malignant and malignant oral disorders

Waseem Jerjes, Zaid Hamdoon, Colin Hopper

Head & Neck Oncology 2012, 4:17

http://www.headandneckoncology.org/content/4/1/17

Expression of Glut-1, HIF-1alpha, PI3K and p-Akt in a case of ceruminous adenoma

Wan-Qin Shen, Ke-Jia Cheng, Yang-Yang Bao, Shui-Hong Zhou and Hong-Tian Yao

Head & Neck Oncology 2012, 4:18

http://www.headandneckoncology.org/content/4/1/18

Systemic therapy in the management of metastatic or advanced salivary gland cancers

Aymen Lagha, Nesrine Chraiet, Mouna Ayadi, Sarra Krimi, Bassem Allani, Hela Rifi, Henda Raies and Amel Mezlini

Head & Neck Oncology 2012, 4:19

http://www.headandneckoncology.org/content/4/1/19

Tongue cancer in young patients: Case report of a 26-year-old patient.

Aleksandra Crede, Michael Locher and Marius Bredell

Head & Neck Oncology 2012, 4:20

http://www.headandneckoncology.org/content/4/1/20

Reconstruction of scalp defects with the radial forearm free flap

Larissa Sweeny, Brendan T Eby, J. Scott Magnuson, William R Carroll and Eben L Rosenthal

Head & Neck Oncology 2012, 4:21

http://www.headandneckoncology.org/content/4/1/21

The use of specific anti-growth factor antibodies to abrogate the oncological consequences of transfusion in head & neck squamous cell carcinoma: an in vitro study

Tahwinder Upile, Waseem Jerjes, Sandeep Singh, Mohammed Al-Khawalde, Zaid Hamdoon, Hani Radhi, Colin Hopper

Head & Neck Oncology 2012, 4:22

http://www.headandneckoncology.org/content/4/1/22

Definitive radiotherapy for early stage glottic cancer by 6 MV photons

Chi-Chung TONG, Kwok-Hung AU, Roger Kai-Cheong NGAN, Sin-Ming CHOW, Foon-Yiu CHEUNG, Yiu-Tung FU, Joseph Siu-Kei AU and Stephen Chun-Key LAW

Head & Neck Oncology 2012, 4:23

http://www.headandneckoncology.org/content/4/1/23

Branchial cysts within the parotid salivary gland

Tahwinder Upile, Waseem Jerjes, Mohammed Al-Khawalde, Panagiotis Kafas, Steve Frampton, Angela Gray, Bruce Addis, Ann Sandison, Nimesh Patel, Holger Sudhoff, Hani Radhi

Head & Neck Oncology 2012, 4:24

http://www.headandneckoncology.org/content/4/1/24

A patient with ulcerated calcifying epithelioma of Malherbe in the pinna: case report

Tahwinder Upile, Waseem Jerjes, Fabian Sipaul, Ann Sandison, Panagiotis Kafas, Mohammed Al-Khawalde, Hani Radhi

Head & Neck Oncology 2012, 4:25

http://www.headandneckoncology.org/content/4/1/25

Computed tomography and pathological findings of five nasal neurilemmomas

Jing Hu, Yang-Yang Bao, Ke-Jia Cheng, Shui-Hong Zhou, Ling-Xiang Ruan and Zhou-Jun Zheng

Head & Neck Oncology 2012, 4:26

http://www.headandneckoncology.org/content/4/1/26

Metastatic rhabdomyosarcoma of the thyroid gland, a case report

Mohamed T Hafez, Mohamed A Hegazy, Khaled Abd Elwahab, Mohammad Arafa, Islam Abdou and Basel Refky

Head & Neck Oncology 2012, 4:27

http://www.headandneckoncology.org/content/4/1/27

Squamous cell carcinoma of the oral cavity and the oropharynx in patients less than 40 years of age: a 20-year analysis

Samuel E Udeabor, Majeed Rana, Gerd Wegener, Nils-Claudius Gellrich and André M Eckardt

Head & Neck Oncology 2012, 4:28

http://www.headandneckoncology.org/content/4/1/28

Structural validation of oral mucosal tissue using optical coherence tomography

Zaid Hamdoon, Waseem Jerjes, Raed Al-Delayme, Gordon McKenzie, Amrita Jay, Colin Hopper

Head & Neck Oncology 2012 4:29

http://www.headandneckoncology.org/content/4/1/29

Solitary giant neurofibroma of the neck subjected to photodynamic therapy: case study

Zaid Hamdoon, Waseem Jerjes, Raed Al-Delayme, Colin Hopper

Head & Neck Oncology 2012 4:30

http://www.headandneckoncology.org/content/4/1/30

Oral sex, cancer and death: sexually transmitted cancers

Tahwinder Upile, Waseem Jerjes, Mohammed Al-Khawalde, Hani Radhi and Holger Sudhoff

Head & Neck Oncology 2012 4:31

http://www.headandneckoncology.org/content/4/1/31

Enhanced patient reported outcome measurement suitable for head and neck cancer follow-up clinics

Naseem Ghazali, Derek Lowe and Simon N Rogers

Head & Neck Oncology 2012, 4:32

http://www.headandneckoncology.org/content/4/1/32

A patient with primary Burkitt's lymphoma of the postnasal space: case report

Tahwinder Upile, Waseem Jerjes, Jesuloba Abiola, Panagiotis Kafas, Ann Sandison, Zaid Hamdoon, Mohammed Al-Khawalde and Hani Radhi

Head & Neck Oncology 2012, 4:33

http://www.headandneckoncology.org/content/4/1/33

Positron emission tomography in the detection of occult primary head and neck carcinoma: a retrospective study

Gabriel G Pereira, Joaquim C Silva and Eurico F Monteiro

Head & Neck Oncology 2012, 4:34

http://www.headandneckoncology.org/content/4/1/34

Granulocyte colony-stimulating factor-producing squamous cell carcinoma of the lower gingiva: a case report

Jun-ichi Kobayashi Dr., Akihiro Miyazaki Dr., Takashi Yamamoto Dr., Kenji Nakamori Dr., Rina Suzuki Dr., Takeshi Kaneko Dr., Naohiro Suzuki Dr. and Hiroyoshi Hiratsuka Prof.

Head & Neck Oncology 2012, 4:35

http://www.headandneckoncology.org/content/4/1/35

Spinal metastasis in head and neck cancer

Gregory M Trilling, Hyongyu Cho, Mohamed A Ugas, Samerah Saeed, Asia Katunda, Waseem Jerjes and Peter V Giannoudis

Head & Neck Oncology 2012, 4:36

http://www.headandneckoncology.org/content/4/1/36

Analysis of the compatibility of dental implant systems in fibula free flap reconstruction

Ramin Carbiner, Waseem Jerjes, Kaveh Shakib, Peter V Giannoudis and Colin Hopper

Head & Neck Oncology 2012, 4:37

http://www.headandneckoncology.org/content/4/1/37

p16 overexpression in malignant and premalignant lesions of the oral and esophageal mucosa following allogeneic hematopoietic stem cell transplantation

Yasumasa Kakei, Masaya Akashi, Hideki Komatsubara, Tsutomu Minamikawa and Takahide Komori

Head & Neck Oncology 2012, 4:38

http://www.headandneckoncology.org/content/4/1/38

Spinal metastasis in thyroid cancer

Sami Ramadan, Mohamed A Ugas, Richard J Berwick, Manisha Notay, Hyongyu Cho, Waseem Jerjes, Peter V Giannoudis

Head & Neck Oncology 2012, 4:39

http://www.headandneckoncology.org/content/4/1/39

Feasibility of recruitment to an oral dysplasia trial in the United Kingdom

Paul C Nankivell, Janet A Dunn, Michael J.S. Langman and Hisham Mehanna

Head & Neck Oncology 2012, 4:40

http://www.headandneckoncology.org/content/4/1/40

Effect of human beta-defensin-3 on head and neck cancer cell migration using micro-fabricated cell islands

Kevin Wang, Joanne H Wang, Harihara Baskaran, Russell Wang and Rick Jurevic

Head & Neck Oncology 2012, 4:41

http://www.headandneckoncology.org/content/4/1/41

